# Maternal disability and initiation and duration of breastfeeding: analysis of a Canadian cross-sectional survey

**DOI:** 10.1186/s13006-023-00608-7

**Published:** 2023-12-21

**Authors:** Hilary K. Brown, Lesley Pablo, Natalie V. Scime, Amira M. Aker, Cindy-Lee Dennis

**Affiliations:** 1 Department of Health & Society, University of Toronto Scarborough, Toronto, ON Canada; 2 Dalla Lana School of Public Health, University of Toronto, Toronto, ON Canada; 3 Women’s College Research Institute, Women’s College Hospital, Toronto, ON Canada; 4 Lawrence S. Bloomberg Faculty of Nursing, Toronto, ON Canada; 5 Lunenfeld-Tannenbaum Research Institute, Mount Sinai Hospital, Toronto, ON Canada; 6 Department of Psychiatry, University of Toronto, Toronto, ON Canada

**Keywords:** Breastfeeding, Cross-sectional studies, Disabled persons, Health equity, Health surveys

## Abstract

**Background:**

The World Health Organization recommends breastfeeding as the best method for infant feeding. Known risk factors for breastfeeding non-initiation and early cessation of breastfeeding are diverse and include low breastfeeding self-efficacy, poverty, smoking, obesity, and chronic illness. Although women with disabilities experience elevated rates of these risk factors, few studies have examined their breastfeeding outcomes. Our objective was to examine breastfeeding non-initiation and early cessation of breastfeeding in women with and without disabilities.

**Methods:**

We used data from the 2017–2018 Canadian Community Health Survey. Included were *n* = 4,817 women aged 15–55 years who had a birth in the last five years, of whom 26.6% had a disability, ascertained using the Washington Group Short Set on Functioning. Prevalence ratios (aPR) of breastfeeding non-initiation, and of early cessation of any and exclusive breastfeeding before 6 months, were calculated for women with versus without disabilities. We also examined disability by severity (moderate/severe and mild, separately) and number of action domains impacted (≥ 2 and 1, separately). The main multivariable models were adjusted for maternal age, marital status, level of education, annual household income level, and immigrant status.

**Results:**

There were no differences between women with and without disabilities in breastfeeding non-initiation (9.6% vs. 8.9%; aPR 0.88, 95% CI 0.63, 1.23). Women with disabilities were more likely to have early cessation of any (44.4% vs. 35.7%) and exclusive breastfeeding before 6 months (66.9% vs. 61.3%), with some attenuation in risk after adjustment for sociodemographic factors (aRR 1.15, 95% CI 0.99, 1.33 and aRR 1.07, 95% 0.98, 1.16, respectively). Disparities were larger for women with moderate/severe disabilities and disabilities in ≥ 2 domains, with differences attenuated by adjustment for socio-demographics.

**Conclusions:**

Women with disabilities, and particularly those with moderate/severe and multiple disabilities, could benefit from tailored, accessible breastfeeding supports that attend to the social determinants of health.

**Supplementary Information:**

The online version contains supplementary material available at 10.1186/s13006-023-00608-7.

## Background

The World Health Organization (WHO) recommends breastfeeding as the best method for infant feeding [1]. Guidelines promote exclusive breastfeeding for the first 6 months, with continued breastfeeding after introduction of complementary foods, up to 2 years and beyond [2]. Benefits of breastfeeding for children include better cognitive outcomes [3]; lower rates of otitis media, respiratory tract infections, and diarrheal morbidity and mortality [4]; and reduced risks of obesity and type 2 diabetes [5]. Breastfeeding is also associated with reduced risks of maternal breast and ovarian cancer, and cardiovascular disease [6, 7]. Yet, despite documented benefits, breastfeeding rates remain low. In North America, for example, 81–91% of women initiate breastfeeding; 62% exclusively breastfeed to 3 months, and only one-third to 6 months [8, 9]. Given the dose-response relationship between duration of exclusive breastfeeding and its benefits [10], there is a need to identify groups for whom tailored breastfeeding supports might help to optimize breastfeeding success.

Known risk factors for breastfeeding non-initiation and early cessation are diverse and include low breastfeeding self-efficacy, poverty, smoking, obesity, and chronic illness [11–13]. There are only a handful of studies on breastfeeding in women with disabilities. These studies suggest women with disabilities are less likely than those without disabilities to report breastfeeding in the immediate postpartum period [14–17]. However, with the focus of studies on breastfeeding initiation, little is known about breastfeeding duration according to WHO guidelines among women with disabilities. This is an important clinical gap given women with physical, sensory, and cognitive disabilities represent nearly 20% of the reproductive-aged population [18] and experience many of the known risk factors for suboptimal breastfeeding outcomes [19, 20]. Qualitative research also suggests they experience significant barriers to breastfeeding, including negative health care provider attitudes, communication barriers, and lack of tailored information and adapted resources [21, 22]. Further research on breastfeeding outcomes in women with disabilities is needed to inform development of breastfeeding supports that meet the needs of this maternal population.

### Aim

Our aim was to examine breastfeeding non-initiation as well as early cessation of any and exclusive breastfeeding among Canadian women with disabilities compared to those without disabilities.

## Methods

### Study design and setting

We used data from the 2017–2018 cycle of the Canadian Community Health Survey (CCHS). The CCHS is a cross-sectional survey comprising a representative sample of Canadians 12 years of age and older, excluding those living in institutions, on Reserves, and in certain regions of Québec, as well as those serving in the Armed Forces (< 3% of the population) [23]. The CCHS used a complex multi-stage sampling design, with the sample ≥ 18 years accrued from the Labour Force Survey sampling frame and the 12 to 17-year-old sample accrued from the Canadian Child Tax Benefits file. Questionnaires were administered using computer-assisted interviews and were 35 to 45 min long. We included all 15 to 55-year-old women who gave birth in the previous five years and had complete data on the CCHS questions of interest. Analyses were conducted at the University of Toronto Research Data Centre; research ethics board approval was not required due to the use of deidentified secondary data.

### Measures

Disability was ascertained using the Washington Group Short Set on Functioning (WG-SS), which uses the WHO’s International Classification of Functioning, Disability, and Health as a framework to collect data on difficulties a person has performing basic universal actions [24]. Questions assess difficulties in six domains—i.e., seeing, hearing, walking or climbing stairs, remembering or concentrating, self-care, and communicating—with response options of “no difficulty”, “some difficulty”, “a lot of difficulty”, and “cannot do at all”. Women were classified as having a disability if they reported “some difficulty”, “a lot of difficulty”, or “cannot do at all” in ≥ 1 domains. Women without a disability were the referent. We further examined disability by severity (i.e., mild or moderate/severe) and the number of domains impacted (i.e., 1 or ≥ 2).

Our main outcomes were: (1) breastfeeding non-initiation, defined by a negative response to the question, “Was [your last child] breastfed or given breastmilk even for a short period of time?” [12]; (2) early cessation of any breastfeeding prior to 6 months in response to the question, “How long did you breastfeed or give breast milk to [your last child]?” [12]; and (3) early cessation of exclusive breastfeeding prior to 6 months in response to questions about the timing of addition of other liquids (i.e., milk, formula, water, juice, tea, or herbal mixture) or solids (i.e., cereals, mashed up or pureed meat, vegetables, or fruits) to the feeds [12]. We also examined cessation of breastfeeding by 12 and 24 months, as secondary, longer-term outcomes.

As covariates, we measured (1) sociodemographic characteristics and (2) health behaviours and comorbidities. Sociodemographic characteristics were maternal age (i.e., 15–24 and 35–55 vs. 25–34 years), marital status (i.e., single, widowed, separated, or divorced, vs. married or common-law), level of education (i.e., high school diploma or less, vs. some post-secondary education or more), annual household income level (i.e., < $40,000, $40,000 to $79,999, vs. ≥ $80,000 CAD), and immigration status (i.e., born outside of vs. in Canada). Health behaviours and comorbidities were smoking status (i.e., daily or occasionally, vs. not at all), body mass index (BMI; i.e., overweight or obese, vs. normal or underweight), and diabetes mellitus and chronic hypertension diagnosed by a health professional (i.e., present, vs. absent).

### Statistical analysis

We described the proportions of women with and without disabilities with each baseline characteristic and compared them using standardized differences [25].

We then used Modified Poisson regression [26] to estimate the prevalence ratios (PR) and corresponding 95% confidence intervals (CI) of breastfeeding non-initiation, early cessation of any breastfeeding by 6 months, and early cessation of exclusive breastfeeding by 6 months, comparing women with and without disabilities. The main multivariable models adjusted for maternal age, marital status, level of education, annual household income level, and immigrant status. Health behaviours and comorbidities that could explain hypothesized disparities between women with and without disabilities (i.e., smoking status, BMI, diabetes, and chronic hypertension) were added to the models in a second step, as these were considered possible pathway variables (Additional file 1).

We conducted several additional analyses. First, we modelled the secondary outcomes of breastfeeding cessation by 12 and 24 months. Second, we examined disability by severity (i.e., moderate/severe and mild disabilities, vs. no disability) and the number of action domains (i.e., ≥ 2 and 1 domains, vs. no disability). Third, we restricted the sample to women who had their baby ≤ 24 months before data collection, to maximize accuracy of breastfeeding recall [27] and reduce the chance that disability onset followed pregnancy. Finally, using descriptive analyses, we compared women with and without disabilities on the reason for non-initiation (“bottle feeding is easier”, “breastfeeding is unappealing”, “medical condition in the mother”, “other”) and early cessation of exclusive breastfeeding (“not enough breast milk”, “baby was ready for solid foods”, “inconvenience or fatigue due to breastfeeding”, “difficulty with breastfeeding”, “medical condition in the mother”, “medical condition in the baby”, “planned to stop at this time”, “child weaned him or herself”, “returned to work or school”, “other”).

All analyses were weighted using a CCHS-assigned weight representing the individual’s contribution to the total population, wherein weights accounted for the CCHS multi-stage sampling design and were adjusted for population projections of age and sex strata within each province as well as survey non-response. The original size of the sample was maintained by dividing individual weights by the average size of the weight in the sample [23].

SAS version 9.4 (SAS Institute Inc., Cary, NC) was used for all analyses.

## Results

Our sample included *n* = 4,817 women aged 15–55 years with a birth in the last 5 years. Among respondents, 26.6% had a disability, with 23.7% of women reporting a mild disability and 2.9% reporting a moderate/severe disability. 19.4% of women reported a disability in only 1 action domain, and 7.2% in ≥ 2 action domains, with the action domains impacted being seeing (10.3%), hearing (3.7%), walking or climbing stairs (4.1%), remembering or concentrating (15.3%), self-care (1.1%), and communicating (2.6%). Compared to women without disabilities, women with disabilities were more likely to have lower educational attainment (high school diploma or less); were less likely to have a high annual income (≥ $80,000 CAD); and were more likely to smoke and be overweight or obese (Table 1).

**Table 1:** Baseline characteristics of the study sample. Data are presented as weighted N (%)

Characteristic	Disability	No disability	Standardized difference
	***N* = 1,282**	***N* = 3,535**	
Age			
15–24 years	126 (9.8)	140 (4.0)	0.06
25–34 years	649 (50.6)	1898 (53.7)	0.03
35–55 years	507 (39.6)	1497 (42.4)	0.03
Single, widowed, separated, or divorced	232 (18.1)	414 (11.7)	0.06
High school diploma or less	429 (33.5)	659 (18.6)	0.15
Annual household income level			
< $40,000 CAD	306 (23.9)	607 (17.2)	0.07
$40,000 to $79,999 CAD	352 (27.4)	852 (24.1)	0.03
≥ $80,000 CAD	624 (48.7)	2076 (58.7)	0.10
Immigrant	333 (26.0)	1221 (34.5)	0.09
Daily or occasional smoker	317 (24.7)	406 (11.5)	0.13
Overweight or obese	680 (53.1)	1495 (42.3)	0.11
Diabetes mellitus	37 (2.9)	76 (2.2)	0.01
Chronic hypertension	42 (3.3)	72 (2.0)	0.01

*Note*: weighted Ns are rounded to the nearest integer

There were no differences in the proportions of women with and without disabilities who did not initiate breastfeeding (9.6% vs. 8.9%; aPR 0.88, 95% CI 0.63, 1.23) (Table 2). Women with moderate/severe disabilities (16.4% vs. 8.9%; PR 1.85, 95% CI 1.05, 3.26) and disabilities in ≥ 2 action domains (14.6% vs. 8.9%; PR 1.64, 95% CI 1.11, 2.43) were more likely than those without disabilities to not initiate breastfeeding; however, point estimates were reduced and 95% CI widened after adjusting for socio-demographics (moderate/severe disabilities: aPR 1.32, 95% CI 0.73, 2.38; disabilities in ≥ 2 domains: aPR 1.22, 95% CI 0.82, 1.81). Associations were further attenuated after adjusting for health behaviours and comorbidities.

**Table 2:** Breastfeeding non-initiation in women with and without disabilities

Exposure definition	N (%) with outcome	PR (95% CI)	aPR (95% CI)^a^	aPR (95% CI)^b^
**Any disability**				
Disability	123 (9.6)	1.08 (0.77, 1.52)	0.88 (0.63, 1.23)	0.80 (0.57, 1.12)
No disability	314 (8.9)	[Referent]	[Referent]	[Referent]
**Disability severity**				
Moderate/severe disability	21 (16.4)	1.85 (1.05, 3.26)	1.32 (0.73, 2.38)	1.14 (0.63, 2.06)
Mild disability	102 (8.8)	0.99 (0.68, 1.45)	0.82 (0.57, 1.20)	0.75 (0.52, 1.09)
No disability	314 (8.9)	[Referent]	[Referent]	[Referent]
**Action domains impacted**				
Disability in ≥ 2 domains	51 (14.6)	1.64 (1.11, 2.43)	1.22 (0.82, 1.81)	1.05 (0.70, 1.56)
Disability in 1 domain	72 (7.7)	0.87 (0.55, 1.38)	0.74 (0.47, 1.17)	0.69 (0.44, 1.08)
No disability	314 (8.9)	[Referent]	[Referent]	[Referent]

*Note*: weighted Ns are rounded to the nearest integer

^a^Adjusted model included maternal age, marital status, level of education, annual household income level, and immigrant status

^b^Adjusted model included maternal age, marital status, level of education, annual household income level, immigrant status, smoking status, BMI, diabetes mellitus, and chronic hypertension

Women with disabilities were more likely than those without disabilities to have early cessation of any breastfeeding before 6 months (44.4% vs. 35.7%; PR 1.24, 95% CI 1.08, 1.14), but the point estimate was reduced, and 95% CI shifted to border the null, after adjusting for socio-demographics (aPR 1.15, 95% CI 0.99, 1.33) (Table 3). Similar patterns were seen in women with moderate/severe disabilities (54.6% vs. 35.7%; aPR 1.36, 95% CI 0.88, 2.11) and disabilities in ≥ 2 domains (54.1% vs. 35.7%; aPR 1.33, 95% CI 1.06, 1.66)—though the latter result remained statistically significant after adjustment for socio-demographics. There were small differences in the likelihood of early cessation of exclusive breastfeeding before 6 months for women with any disability (66.9% vs. 61.3%; PR 1.09, 95% CI 1.01, 1.18) and disabilities in ≥ 2 domains (70.9% vs. 61.3%; PR 1.16, 95% CI 1.02, 1.31); again, point estimates were reduced, and 95% CI shifted to border the null, after adjustment for socio-demographics (any disability: aPR 1.07, 95% CI 0.98, 1.16; disabilities in ≥ 2 domains: aPR 1.12, 95% CI 0.98, 1.28). Results were attenuated after further adjusting for health behaviours and comorbidities.

**Table 3:** Early cessation of any and exclusive breastfeeding before 6 months in women with and without disabilities

Outcome	Exposure definition	N (%) with outcome	PR (95% CI)	aPR (95% CI)^a^	aPR (95% CI)^b^
**Early cessation of any breastfeeding before 6 months**^**c**^	**Any disability**				
	Disability	393 (44.4)	1.24 (1.08, 1.44)	1.15 (0.99, 1.33)	1.08 (0.93, 1.25)
	No disability	856 (35.7)	[Referent]	[Referent]	[Referent]
	**Disability severity**				
	Moderate/severe disability	54 (54.6)	1.53 (1.10, 2.12)	1.36 (0.88, 2.11)	1.21 (0.81, 1.82)
	Mild disability	339 (43.1)	1.21 (1.04, 1.41)	1.12 (0.96, 1.30)	1.06 (0.92, 1.23)
	No disability	856 (35.7)	[Referent]	[Referent]	[Referent]
	**Action domains impacted**				
	Disability in ≥ 2 domains	130 (54.1)	1.51 (1.25, 1.84)	1.33 (1.06, 1.66)	1.18 (0.95, 1.46)
	Disability in 1 domain	263 (40.8)	1.14 (0.96, 1.36)	1.08 (0.91, 1.28)	1.04 (0.88, 1.23)
	No disability	856 (35.7)	[Referent]	[Referent]	[Referent]
**Early cessation of exclusive breastfeeding before 6 months**^**d, e**^	**Any disability**				
	Disability	720 (66.9)	1.09 (1.005, 1.18)	1.07 (0.98, 1.16)	1.04 (0.96, 1.13)
	No disability	1773 (61.3)	[Referent]	[Referent]	[Referent]
	**Action domains impacted**				
	Disability in ≥ 2 domains	200 (70.9)	1.16 (1.02, 1.31)	1.12 (0.98, 1.28)	1.06 (0.93, 1.21)
	Disability in 1 domain	520 (65.5)	1.07 (0.97, 1.17)	1.05 (0.95, 1.15)	1.03 (0.94, 1.13)
	No disability	1773 (61.3)	[Referent]	[Referent]	[Referent]

*Note*: weighted Ns are rounded to the nearest integer

^a^Adjusted model included maternal age, marital status, level of education, annual household income level, and immigrant status

^b^Adjusted model included maternal age, marital status, level of education, annual household income level, immigrant status, smoking status, BMI, diabetes mellitus, and chronic hypertension

^c^Analysis restricted to *N* = 3,282 women who reported ever breastfeeding, excluding those who were still breastfeeding at the time of the interview

^d^Analysis restricted to *N* = 3,967 women who reported ever breastfeeding, excluding those who had not yet added any other liquid or solid foods to their baby's feeds at the time of the interview

^e^Results for disability severity could not be reported due to small differences in sample sizes compared to other analyses

There were no meaningful differences between women with and without disabilities, overall or by disability severity or the number of domains impacted by the disability, in terms of breastfeeding cessation by 12 or 24 months (Additional file 2).

Findings were similar when we restricted the sample to women who had their baby ≤ 24 months before data collection, to maximize accuracy of maternal recall of breastfeeding and reduce the chance that disability onset followed pregnancy (Additional file 3).

Finally, there were few differences between the groups in reasons for breastfeeding non-initiation and early cessation of exclusive breastfeeding (Additional file 4). However, women with disabilities were more likely than those without disabilities to list social and other factors (e.g., “breastfeeding is unappealing”, “returned to work or school”) and were less likely to list medical factors (e.g., “medical condition of the mother”) as reasons.

## Discussion

### Summary of findings

In this large, nationally representative cross-sectional survey, we found no meaningful difference between women with and without disabilities in rates of breastfeeding non-initiation, and an increased likelihood in women with disabilities of early cessation of any and exclusive breastfeeding by 6 months that was largely explained by sociodemographic factors, health behaviours, and comorbidities. Disparities increased with greater disability severity and number of disability domains impacted; however, despite moderate effect sizes, most findings had wide confidence intervals that bordered the null. There were no notable differences between groups in longer-term breastfeeding duration.

### Comparison to previous research

Only four quantitative studies have investigated breastfeeding in disabled women. A UK survey by the Care Quality Commission of the English National Health Service Trusts found 70% of women with disabilities compared to 79% of those without disabilities breastfed in the first few days postpartum [14]. A US study using data from the Rhode Island Pregnancy Risk Assessment Monitoring System found 70% of women with disabilities who had recently given birth reported breastfeeding or pumping (vs. 75% in those without disabilities), and 45% were currently breastfeeding (vs. 53% in those without disabilities) [15]. Another US study using the Massachusetts Pregnancy to Early Life Longitudinal dataset found a rate of breastfeeding at hospital discharge of 49% in women with cognitive disabilities, versus 74% in women with diabetes mellitus and 77% in those with neither condition [16]. Finally, a study using health administrative data in Ontario, Canada, found women with cognitive and multiple disabilities were less likely than those without disabilities to have any and exclusive breastfeeding at hospital discharge [17]. While we found no differences in rates of breastfeeding non-initiation in women with and without disabilities overall, our data add to this literature by suggesting women with disabilities, and particularly those with moderate/severe and multiple disabilities, were more likely to stop breastfeeding early, and that these disparities were largely driven by other social and health factors.

### Explanation for findings

Low rates of breastfeeding non-initiation in women with and without disabilities overall may reflect widespread initial in-hospital supports for breastfeeding under the Canadian adaptation of the WHO’s Baby Friendly Hospital Initiative [28, 29].

However, disparities in breastfeeding duration warrant investigation. Differences in rates of early breastfeeding cessation between women with and without disabilities were reduced after adjusting for sociodemographic factors. Disabled women have higher rates of poverty and greater barriers to education than those without disabilities [19, 20]. These are known barriers to breastfeeding, via lower health literacy, reduced access to breastfeeding supports, and a need to return to work early [30]. Indeed, one-quarter of women with disabilities in our sample (vs. 16.9% of women without disabilities) endorsed “returned to work or school” as a reason for early cessation of exclusive breastfeeding, supporting the hypothesis that social factors play an important role. Adjustment for health behaviours and comorbidities further attenuated associations. Nicotine exposure, increased adipose tissue, and chronic illness are associated with physiologic changes that interfere with milk production [31, 32], and may impact breastfeeding outcomes for women with disabilities given the higher prevalence of these risk factors in this population.

Other unmeasured factors may also be important, including perinatal complications [33–35]. Qualitative studies also suggest disability-related symptoms such as pain and fatigue [22], receipt of conflicting advice about breastfeeding while using medications [36], and difficulty accessing tailored information and adaptive strategies to support breastfeeding [21, 22] may act as barriers. However, interestingly, women with disabilities were no more likely to list “medical condition in the mother” as a reason for breastfeeding non-initiation or early cessation, suggesting these disability-related factors may not have been major contributors to breastfeeding outcomes in our sample. Finally, women with disabilities may have different breastfeeding intentions than those without disabilities, and may have planned to breastfeed for shorter periods of time. For example, our data suggest women with disabilities were more likely than those without disabilities to describe breastfeeding as unappealing. Future studies should examine barriers to breastfeeding for women with disabilities in further detail.

### Strengths and limitations

Strengths of our study include the use of the WG-SS [23] to measure disability. The WG-SS is used widely in Censuses and surveys internationally, and acknowledges that disability represents the intersection between an individual’s functional limitations and environmental barriers that limit participation [24]. Our study is limited by the cross-sectional nature of the CCHS. It is possible a disability could have been acquired after the birth, rather than before. However, our findings suggest this is not the case. For example, there was a dose-response relation between the severity of disability, as well as the number of action domains, and breastfeeding outcomes. The cross-sectional nature of the CCHS also means recall bias could have impacted women’s responses to questions about breastfeeding. Studies have shown a high degree of accuracy of breastfeeding duration recall even 20 years later [37]. However, recall of the timing of introduction of other liquids and solids is less reliable [37]. Nevertheless, our findings were similar when limited to a smaller sample with a birth in the last 2 years. It may be difficult for people with disabilities, particularly cognitive and communication disabilities, to participate in lengthy surveys such as the CCHS [38]. It is therefore likely that the sample captures mostly mild disabilities; we may have thus under-estimated disparities experienced by all women with disabilities. We had no information on covariates such as parity, or possible explanatory factors such as perinatal complications [35]. We also had no data on breastfeeding supports, breastfeeding self-efficacy, or breastfeeding intentions [39, 40]; these are important areas for research to understand the breastfeeding experiences of disabled women.

## Conclusions

Our findings have several implications. The finding that sociodemographic factors, along with health behaviours and comorbidities, largely explained disparities in breastfeeding duration between women with and without disabilities is notable. It indicates that these factors could be targets for preconception health promotion efforts supporting later breastfeeding in women with disabilities. Our findings also suggest the need to ensure that prenatal education that supports development of breastfeeding knowledge and self-efficacy, normalizes breastfeeding, and dispels negative myths is accessible to and responsive to the needs of women with disabilities [11, 41]. There may also be a need for early access to tailored breastfeeding supports for women with moderate/severe and multiple disabilities in particular, that address social and structural barriers to breastfeeding success. Targeted breastfeeding interventions have been found to improve breastfeeding outcomes in other high-risk groups, including women experiencing poverty and racism and those with perinatal complications or chronic illness [42–45]; the effectiveness of targeted breastfeeding interventions in women with disabilities should also be evaluated. More broadly, health care providers who support breastfeeding, such as pediatricians, public health nurses, and lactation consultants, should receive training on disability, accessible communication, how to adapt breastfeeding techniques, and the multiple ways in which disability intersects with the social determinants of health [17, 21]. Notably, given the socioeconomic risks disproportionately experienced by women with disabilities, and the importance of these factors in explaining breastfeeding outcomes in this group, supports should be freely available and delivered in a way that is mindful of other barriers related to transportation and other factors. Such supports are needed to optimize breastfeeding outcomes in women with disabilities.

### Supplementary Information

**Additional file 1.** Directed acyclic graph showing the relationship between maternal disability status and breastfeeding outcomes.

**Additional file 2.** Cessation of any breastfeeding before 12 and 24 months in women with and without disabilities.

**Additional file 3.** Breastfeeding non-initiation and early cessation of any and exclusive breastfeeding by 6 months in women with and without disabilities, including only women with a birth within ≤ 24 months before their interview date.

**Additional file 4: eTable 4.** Reasons for breastfeeding non-initiation and early cessation of breastfeeding among women with and without disabilities.

## Data Availability

The datasets presented in this article are not readily available because data privacy and confidentiality are protected by the Canadian Statistics Act. Requests to access the datasets should be directed to Canadian Research Data Centre Network (CRDCN) (https://crdcn.org/data).

## Abbreviations

CCHS: Canadian Community Health Survey
CI: Confidence Interval
PR: Prevalence ratio
WHO: World Health Organization
