# Supplementary material for: Maternal disability and initiation and duration of breastfeeding: analysis of a Canadian cross-sectional survey
Source: Int Breastfeed J. 2023 Dec 21;18:70. doi: 10.1186/s13006-023-00608-7 (PMC10734132; doi:10.1186/s13006-023-00608-7)
Supplement: Supplementary file 1 — Additional file 1. Directed acyclic graph showing the relationship between maternal disability status and breastfeeding outcomes. [file 13006_2023_608_MOESM1_ESM.docx]

**Additional file 1. Directed acyclic graph showing the relationship between maternal disability status and breastfeeding outcomes.**

**
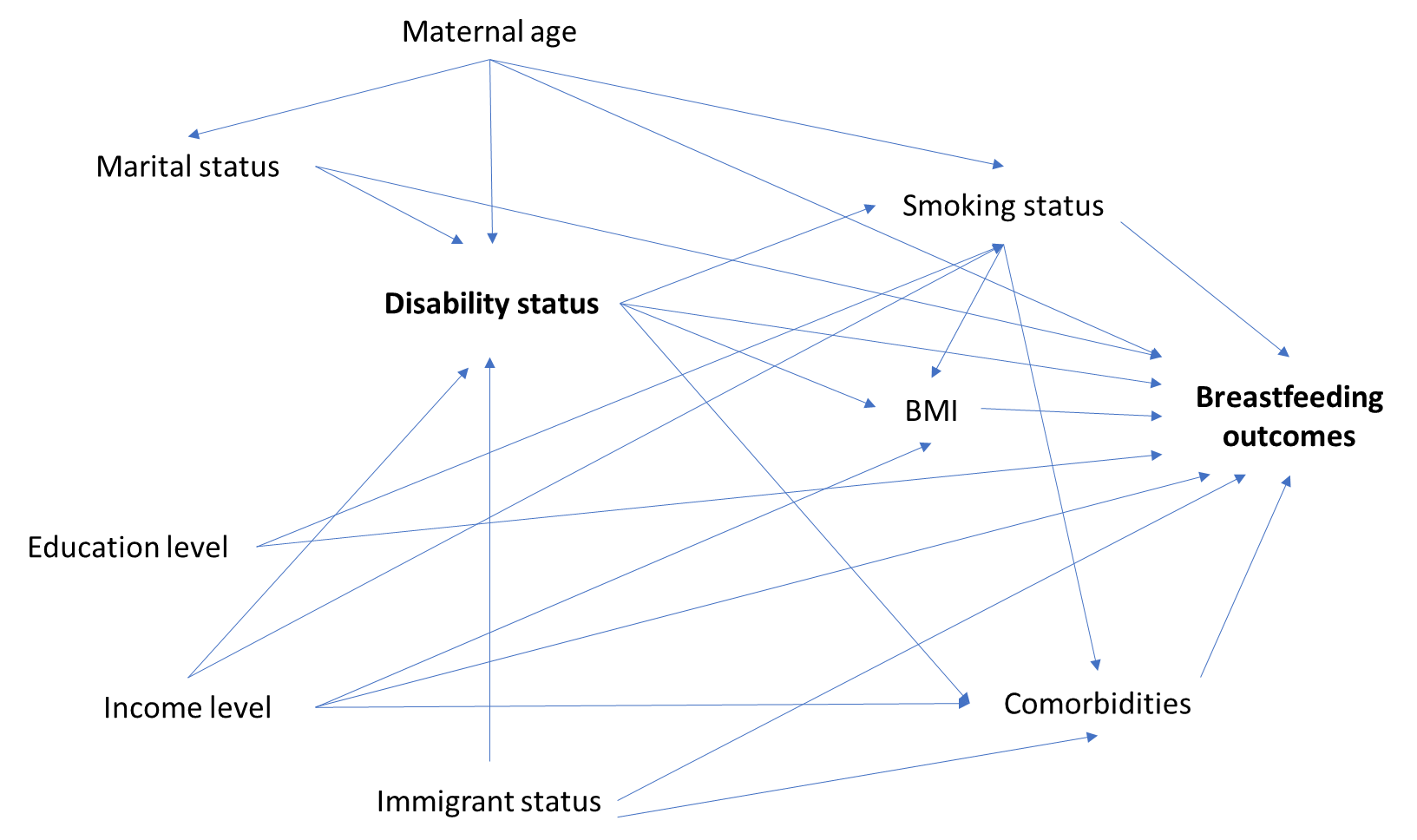
**
