# Supplementary material for: Maternal disability and initiation and duration of breastfeeding: analysis of a Canadian cross-sectional survey
Source: Int Breastfeed J. 2023 Dec 21;18:70. doi: 10.1186/s13006-023-00608-7 (PMC10734132; doi:10.1186/s13006-023-00608-7)
Supplement: Supplementary file 2 — Additional file 2. Cessation of any breastfeeding before 12 and 24 months in women with and without disabilities. [file 13006_2023_608_MOESM2_ESM.docx]

**Additional File 2. Cessation of any breastfeeding before 12 and 24 months in women with and without disabilities.**

| **Outcome** | **Exposure definition** | **N (%) with outcome** | **PR**  **(95% CI)** | **aPR**  **(95% CI)^a^** | **aPR**  **(95% CI)^b^** |
| --- | --- | --- | --- | --- | --- |
| **Cessation of any breastfeeding before 12 months^c^** | **Any disability** |  |  |  |  |
|  | Disability | 611 (69.1) | 1.08 (0.99, 1.17) | 1.04 (0.95, 1.13) | 1.01 (0.93, 1.11) |
|  | No disability | 1539 (64.2) | [Referent] | [Referent] | [Referent] |
|  | **Disability severity** |  |  |  |  |
|  | Moderate/severe disability | 74 (74.8) | 1.17 (0.93, 1.47) | 1.11 (0.83, 1.47) | 1.05 (0.80, 1.39) |
|  | Mild disability | 537 (68.4) | 1.07 (0.97, 1.17) | 1.03 (0.94, 1.12) | 1.01 (0.92, 1.10) |
|  | No disability | 1539 (64.2) | [Referent] | [Referent] | [Referent] |
|  | **Action domains impacted** |  |  |  |  |
|  | Disability in ≥ 2 domains | 173 (72.0) | 1.12 (0.98. 1.28) | 1.05 (0.91, 1.22) | 1.01 (0.87, 1.16) |
|  | Disability in 1 domain | 438 (68.0) | 1.06 (0.96, 1.17) | 1.03 (0.93, 1.14) | 1.02 (0.92, 1.12) |
|  | No disability | 1539 (64.2) | [Referent] | [Referent] | [Referent] |
|  |  |  |  |  |  |
| **Cessation of any breastfeeding before 24 months^c,d^** | **Any disability** |  |  |  |  |
|  | Disability | 814 (92.0) | 0.99 (0.95, 1.03) | 0.99 (0.95, 1.02) | 0.98 (0.95, 1.02) |
|  | No disability | 2231 (93.1) | [Referent] | [Referent] | [Referent] |
|  | **Action domains impacted** |  |  |  |  |
|  | Disability in ≥ 2 domains | 220 (91.4) | 0.98 (0.90, 1.07) | 0.98 (0.91, 1.05) | 0.97 (0.91, 1.05) |
|  | Disability in 1 domain | 594 (92.2) | 0.991 (0.95, 1.03) | 0.99 (0.95, 1.03) | 0.99 (0.95, 1.03) |
|  | No disability | 2231 (93.1) | [Referent] | [Referent] | [Referent] |

Note: weighted N’s are rounded to the nearest integer.

^a^ Adjusted model included maternal age, marital status, level of education, annual household income level, and immigrant status.

^b^ Adjusted model included maternal age, marital status, level of education, annual household income level, immigrant status, smoking status, BMI, diabetes mellitus, and chronic hypertension.

^c^ Analysis restricted to N=3,282 women who reported ever breastfeeding, excluding those who were still breastfeeding at the time of the interview.

^d^ Results for disability severity could not be reported due to small differences in sample sizes compared to other analyses.
