# Supplementary material for: Maternal disability and initiation and duration of breastfeeding: analysis of a Canadian cross-sectional survey
Source: Int Breastfeed J. 2023 Dec 21;18:70. doi: 10.1186/s13006-023-00608-7 (PMC10734132; doi:10.1186/s13006-023-00608-7)
Supplement: Supplementary file 3 — Additional file 3. Breastfeeding non-initiation and early cessation of any and exclusive breastfeeding by 6 months in women with and without disabilities, including only women with a birth within ≤ 24 months before their interview date. [file 13006_2023_608_MOESM3_ESM.docx]

**Additional file 3. Breastfeeding non-initiation and early cessation of any and exclusive breastfeeding by 6 months in women with and without disabilities, including only women with a birth within ≤ 24 months before their interview date.**

| **Outcome** | **Exposure definition** | **% with outcome** | **PR**  **(95% CI)** | **aPR**  **(95% CI)^a^** | **aPR**  **(95% CI)^b^** |
| --- | --- | --- | --- | --- | --- |
| **Breastfeeding non-initiation** | **Any disability** |  |  |  |  |
|  | Disability | 48 (8.6) | 1.36 (0.88, 2.11) | 1.06 (0.71, 1.59) | 0.97 (0.64, 1.46) |
|  | No disability | 104 (6.3) | [Referent] | [Referent] | [Referent] |
|  |  |  |  |  |  |
| **Early cessation of any breastfeeding before 6 months^c^** | **Any disability** |  |  |  |  |
|  | Disability | 177 (66.3) | 1.33 (1.12, 1.59) | 1.28 (1.07, 1.52) | 1.21 (1.01, 1.44) |
|  | No disability | 402 (49.8) | [Referent] | [Referent] | [Referent] |
|  |  |  |  |  |  |
| **Early cessation of exclusive breastfeeding before 6 months^d^** | **Any disability** |  |  |  |  |
|  | Disability | 330 (72.4) | 1.10 (0.98, 1.23) | 1.07 (0.96, 1.20) | 1.04 (0.93, 1.17) |
|  | No disability | 836 (65.9) | [Referent] | [Referent] | [Referent] |

Note: weighted N’s are rounded to the nearest integer.

^a^ Adjusted model included maternal age, marital status, level of education, annual household income level, and immigrant status.

^b^ Adjusted model included maternal age, marital status, level of education, annual household income level, immigrant status, smoking status, BMI, diabetes mellitus, and chronic hypertension.

^c^ Analysis restricted to N=1,074 women who reported ever breastfeeding, excluding those who were still breastfeeding at the time of the interview.

^d^ Analysis restricted to N=1,724 women who reported ever breastfeeding, excluding those who had not yet added any other liquid or solid foods to their baby's feeds at the time of the interview.
