# Supplementary material for: Maternal disability and initiation and duration of breastfeeding: analysis of a Canadian cross-sectional survey
Source: Int Breastfeed J. 2023 Dec 21;18:70. doi: 10.1186/s13006-023-00608-7 (PMC10734132; doi:10.1186/s13006-023-00608-7)
Supplement: Supplementary file 4 — Additional file 4: eTable 4. Reasons for breastfeeding non-initiation and early cessation of breastfeeding among women with and without disabilities. [file 13006_2023_608_MOESM4_ESM.docx]

**eTable 4. Reasons for breastfeeding non-initiation and early cessation of breastfeeding among women with and without disabilities.** Data are presented as weighted %.

| **Outcome** | **Reasons** | **Disability** | **No disability** | **Standardized difference** |
| --- | --- | --- | --- | --- |
| **Breastfeeding non-initiation** | Bottle feeding is easier | 18 (12.6) | 50 (13.7) | 0.03 |
|  | Breastfeeding is unappealing | 36 (25.4) | 61 (16.9) | 0.21* |
|  | Medical condition in the mother | 51 (35.4) | 148 (40.8) | 0.11* |
|  | Other^a^ | 38 (26.6) | 104 (28.6) | 0.04 |
|  |  |  |  |  |
| **Early cessation of exclusive breastfeeding** | Not enough breast milk | 172 (42.7) | 385 (44.0) | 0.03 |
|  | Baby was ready for solid foods | 46 (11.3) | 59 (6.7) | 0.16* |
|  | Inconvenience or fatigue | 64 (15.9) | 135 (15.5) | 0.01 |
|  | Difficulty with breastfeeding | 34 (8.5) | 72 (8.3) | 0.01 |
|  | Medical condition in the mother | 24 (6.0) | 60 (6.9) | 0.04 |
|  | Medical condition in the baby | 33 (8.3) | 59 (6.7) | 0.06 |
|  | Planned to stop at this time | 30 (7.4) | 105 (12.0) | 0.16* |
|  | Child weaned him or herself | 18 (12.6) | 50 (13.7) | 0.03 |
|  | Returned to work or school | 36 (25.4) | 61 (16.9) | 0.21* |
|  | Other | 51 (35.4) | 148 (40.8) | 0.11* |

Note: weighted N’s are rounded to the nearest integer.

^a^ Includes “Formula is as good as breast milk”.

* Meaningful differences between women with and without disabilities, i.e., with standardized differences > 0.10.
